# A newborn screening pilot study using methylation-sensitive high resolution melting on dried blood spots to detect Prader-Willi and Angelman syndromes

**DOI:** 10.1038/s41598-020-69750-0

**Published:** 2020-08-03

**Authors:** Igor Ribeiro Ferreira, Régis Afonso Costa, Leonardo Henrique Ferreira Gomes, Wilton Darleans dos Santos Cunha, Latife Salomão Tyszler, Silvia Freitas, Juan Clinton Llerena Junior, Zilton Farias Meira de Vasconcelos, Robert D. Nicholls, Letícia da Cunha Guida

**Affiliations:** 1 Laboratório de Alta Complexidade, Instituto Nacional da Saúde da Mulher, da Criança E Do Adolescente Fernandes Figueira, Fiocruz, Avenida Rui Barbosa 716, Flamengo, Rio de Janeiro, RJ 22250-020 Brazil; 2 Instituto Estadual de Diabetes E Endocrinologia Luiz Capriglione (IEDE), Rio de Janeiro, Brazil; 3 Departamento de Genética, Instituto Nacional da Saúde da Mulher, da Criança E Do Adolescente Fernandes Figueira, Fiocruz, Rio de Janeiro, Brazil; 4 Division of Medical Genetics, Department of Pediatrics, UPMC Children’s Hospital of Pittsburgh, University of Pittsburgh, Pittsburgh, PA USA

**Keywords:** Epigenetics, Epigenetics analysis, Imprinting

## Abstract

Prader-Willi (PWS) and Angelman (AS) syndromes are two clinically distinct imprinted disorders characterized by genetic abnormalities at 15q11-q13. Early diagnosis of both syndromes provides improved treatment and accurate genetic counseling. Whole blood (WB) is the most common DNA source of many methodologies to detect PWS and AS, however, the need of WB makes a massive screening difficult in newborns due to economic and technical limitations. The aim of this study was to adapt a Methylation-sensitive High-Resolution Melting (MS-HRM) approach from dried blood spot (DBS) samples, assessing the different DNA isolation techniques and diagnostic performance. Over a 1-year period, we collected 125 DBS cards, of which 45 had already been diagnosed by MS-HRM (20 PWS, 1 AS, and 24 healthy individuals). We tested three different DBS-DNA extraction techniques assessing the DNA concentration and quality, followed by MS-HRM and statistical comparison. Each DBS-DNA extraction method was capable of accuracy in detecting all PWS and AS individuals. However, the efficiency to detect healthy individuals varied according to methodology. In our experience, DNA extracted from DBS analyzed by the MS-HRM methodology provides an accurate approach for genetic screening of imprinting related disorders in newborns, offering several benefits compared to traditional whole blood methods.

## Introduction

Prader-Willi (PWS) and Angelman (AS) syndromes are complex disorders arising from genetic abnormalities in chromosome 15. Both syndromes are considered rare due to the estimated prevalence of 1 in 10,000–30,000 individuals^1,2^. While they occur in the same genomic region, multiple genetic alterations and very distinct clinical characteristics are present. The main features associated with PWS are severe neonatal hypotonia, short stature, small hands and feet, dysmorphic face, early onset of hyperphagia, development of morbid obesity, hypogonadism, and cognitive impairment^3^. Congenital hypotonia represents a diagnostic challenge, especially in newborns, because it is present in several disorders, as metabolic diseases, acute or chronic illness, genetic syndromes, endocrinopathies, myopathies, and central or peripheral nervous system abnormalities^4^. AS patients present delayed psychomotor development, severe mental retardation, absence of speech, seizures, motor oddities, and epilepsy^5^.

There are multiple genetic mechanisms that can lead to PWS or AS. The most frequent occurrence in both syndromes is deletions at the chromosome 15q11-q13 region, affecting approximately 65–75% of individuals with PWS or AS. Deletions on the paternal allele result in PWS, whereas on the maternal allele cause AS^6,7^. Maternal Uniparental Disomy (mUPD) of chromosome 15 is found in about 25% of PWS patients^8,9^, while Paternal Uniparental Disomy (pUPD) occurs in only 3–7% of individuals with AS^2,10^. About 1–5% of cases of both syndromes present hypermethylation (PWS) and hypomethylation (AS) due to an imprinting defect. In 10–15% of these cases the imprinting defect is due to an imprinting centre deletion^11,12^. The remaining AS cases (10–20%) involve coding mutations in the *UBE3A* gene^13,14^.

The diagnosis of PWS and AS depends on a combination of clinical features, molecular studies, and cytogenetic analysis. The most sensitive laboratory approach for the diagnosis of both syndromes is to analyze the DNA methylation pattern of the promoter-exon 1 region of the *SNURF*-*SNRPN* bicistronic gene (15q11.2)^15,16^. Further molecular analysis by Multiplex Ligation Probe-Dependent Amplification (MLPA) and Microsatellite Analysis, along with molecular cytogenetic analysis by Fluorescence in Situ Hybridization (FISH) will reveal the PWS/AS etiology^17,18^. DNA Methylation analysis by Methylation-specific PCR (MS-PCR) technique is based on bisulfite conversion of DNA, followed by PCR amplification with two pairs of primers amplifying paternal and maternal alleles along with agarose gel electrophoresis^15^. However, there is a high risk of false-positives obtained by cross annealing of primers, and the occurrence of false-negatives due to competition for reagents as already reported^19^.

Recently, our group described a methodology using Methylation-sensitive High-Resolution Melting (MS-HRM) where a single pair of primers amplifies the promoter-exon 1 region of the *SNURF*-*SNRPN* locus revealing its methylation status. The MS-HRM approach dispenses additional techniques such as agarose gel electrophoresis to detect positive cases. However, the proposed approach does not provide specific information about the disease etiology requiring subsequent techniques such as FISH, MLPA, and microsatellite analysis to detect the underlying molecular or cytogenetic cause (deletion, uniparental disomy or imprinting defect)^16^.

Dried blood spot (DBS) is a form of collection and storage of blood on a filter paper, called Guthrie cards. These samples contain on average 50 μL of blood per spot and are routinely collected in the first 48–120 h of life as part of the newborn screening programs (NBS) in many countries^20^. The major goal of NBS is to identify treatable inherited diseases, avoiding morbidity, and mortality associated with genetic disorders^21^. Furthermore, DBS collection is simple to perform, requires minimal training, and does not offer biohazard risks to health care workers. Guthrie cards can easily be transported from isolated regions to reference centers, avoiding geographical barriers that would prevent nationwide disease screening. Ultimately, DBS can be easily stored providing the opportunity to perform population studies for incidence and prevalence^22,23^.

This work aimed to assess the feasibility and accuracy of PWS/AS screening on DBS samples, using our previously reported MS-HRM method comparing three different DNA extraction methods and using peripheral whole blood (WB) samples as reference.

## Results

**Figure. 1:**
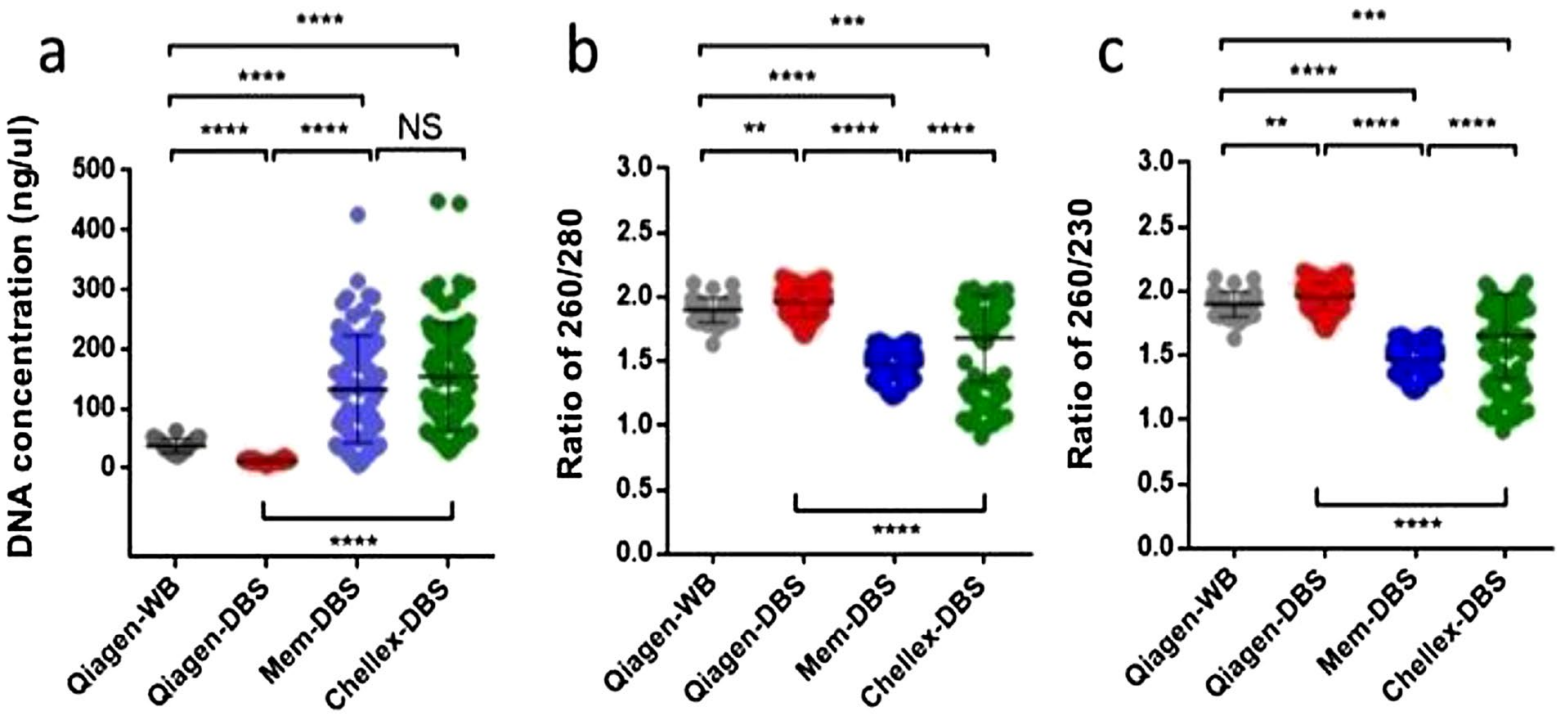
Comparison of DNA quality between nucleic acid extraction methods. The DNA concentration (**a**), and DNA purity were assessed using 260/280 and 260/230 parameters (**b**,**c**). NS *P* > 0.05; **P < 0.01; ***P < 0.001; ****P < 0.0001.

To accomplish the MS-HRM methodology as a potential newborn screening strategy for PWS and AS, this study started by assessing the best DNA extraction for DBS samples. Genomic DNA was isolated from DBS with three different methods (Qiagen-DBS, Mem-DBS, and Chellex-DBS). Initially, the DNA extraction with Mem-DBS and Chellex-DBS kits provided a significantly higher DNA concentration (*P* < 0.0001) compared to the DNA extraction from Qiagen-DBS (Fig. 1a). However, when DNA quality was assessed by the 260/280 wavelength (for protein, phenol, or other contaminants), the Qiagen-DBS strategy showed a significantly higher DNA purity compared to the DNA obtained from Mem-DBS and Chellex-DBS (*P* < 0.0001) (Fig. 1b). In addition, analyzing the 230/260 absorbance (for EDTA, carbohydrates, and phenol contamination), the genomic DNA obtained by the Qiagen-DBS methodology also reached significantly better quality values than Mem-DBS and Chellex-DBS (Fig. 1c) (*P* < 0.0001).

To ensure the DNA quality of all clinical samples (WB or DBS) from each individual the human gene *RPP38* was tested. The amplification curves for *RPP38* for all DNA extraction protocols exclude the possibility of false negatives.

**Figure 2:**
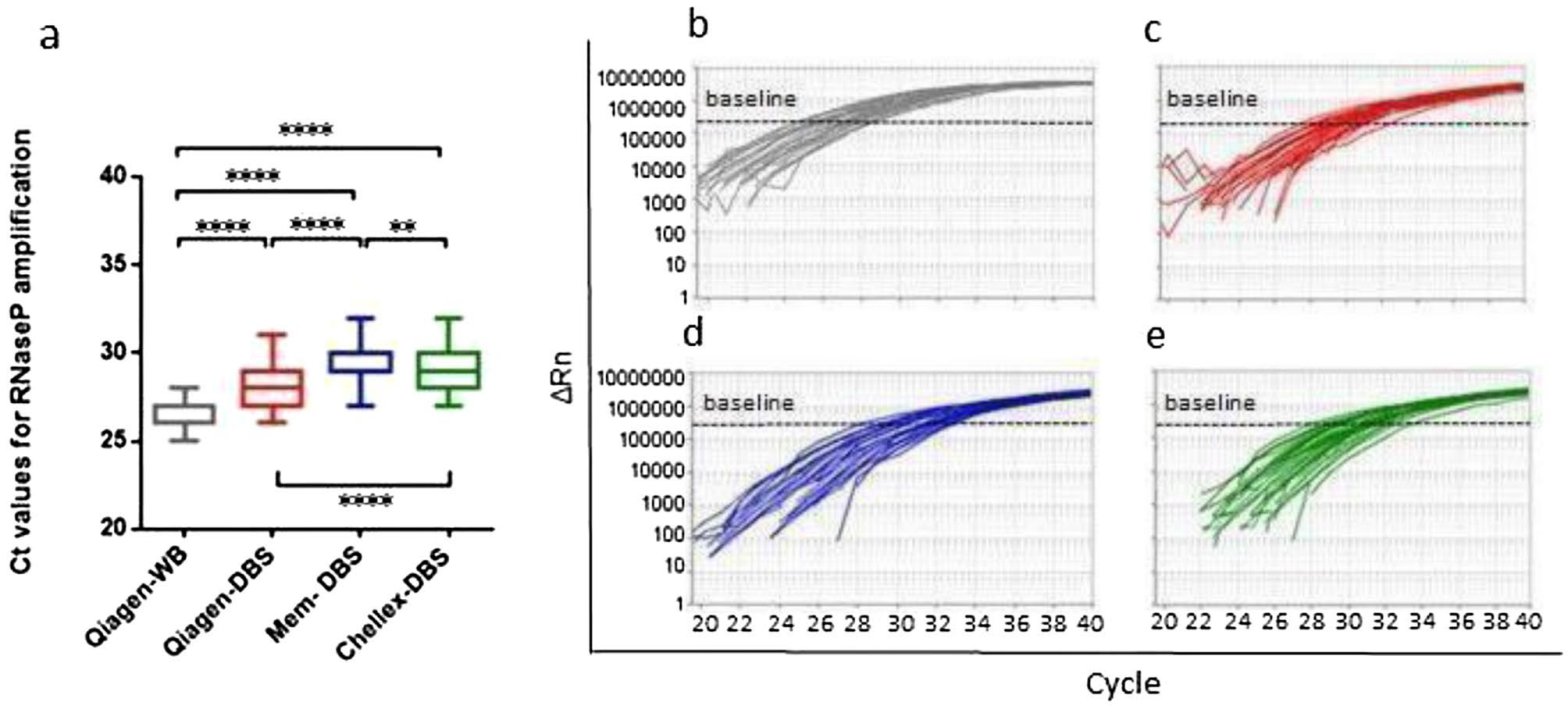
Variations of *RPP38* amplification per DNA extraction method. (**a**) Comparative Ct amplification analysis ****P < 0.0001, **P < 0.01. Representative *RPP38* amplification plots: (**b**) Qiagen-WB; (**c**) Qiagen-DBS; (**d**) Mem-DBS; (**e**) Chellex-DBS.

The Qiagen-DBS amplification curves displayed a mean Ct of 28.18 with a range between 26 and 31, while Mem-DBS and Chellex-DBS presented higher amplification mean Ct of 29.53^27–32^ and 29.03^27–32^, respectively (Fig. 2a and Additional file 1: Table S1). The representative *RPP38* amplification curves are presented in Fig. 2b–e.

The genomic DNA obtained from different clinical samples (WB or DBS) and distinct nucleic acid extraction approaches were treated with bisulfite. Converted DNA was quantified and the recovery concentration ratio was ~ 10 ng/μL (about 50% of the initial DNA input) in all methods.

The MS-HRM methodology previously described by our group was used to amplify the bisulfite modified DBS-DNA^16^. From 125 samples processed with the Qiagen-DBS methodology, 123 samples were amplified (20 PWS, 1 AS, and 102 Healthy individuals) with a mean Ct of 28, consistent with the mean Ct from *RPP38* amplification.

From the Mem-DBS method, only 103 samples were amplified (20 PWS, 1 AS, and 82 Healthy individuals) with a Ct mean of 31. 115 samples from the Chellex-DBS extraction method were amplified (20 PWS, 1 AS, and 94 Healthy individuals) with a mean Ct of 32. These results are consistent with the DNA purity extracted by each different methodology.

**Figure 3:**
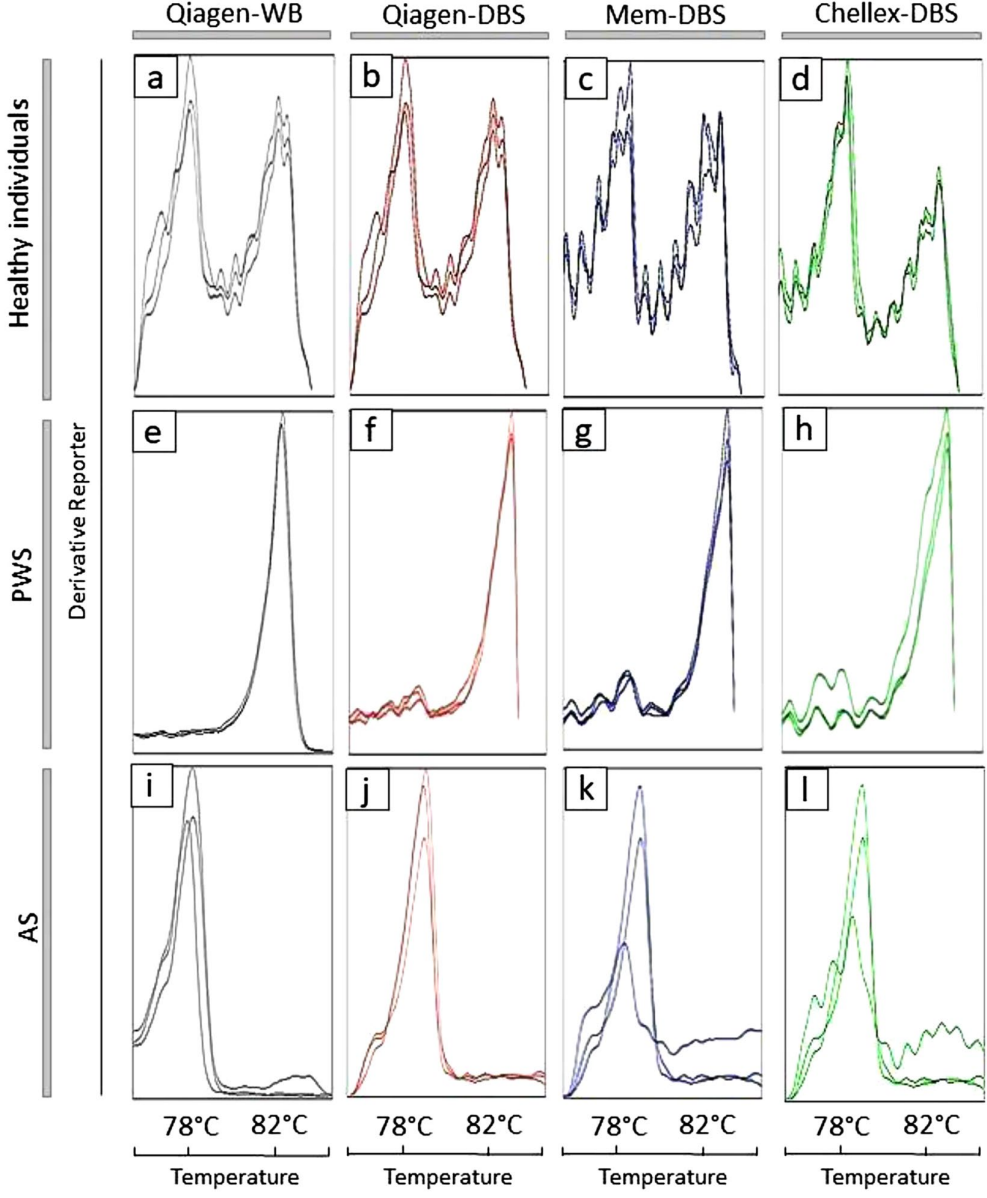
Dissociation curve analysis according to each DNA extraction method. (**a**–**d**) Normal methylation pattern, unmethylated paternal (78 °C), and methylated maternal alleles (82 °C) present. (**e**–**h**) Absence of paternal allele, only the maternal allele is present, confirming the PWS pattern. (**i**–**l**) Absence of the maternal allele, only the paternal allele is present, confirming AS pattern. Dissociation curve analysis per DNA extraction method.

None of the extraction methods changed significantly the melting temperature curve displayed after bisulfite-treated DNA amplification. Among the 45 individuals analyzed by MS-HRM methodology from Qiagen-WB, all 24 healthy individuals, 20 Prader-Willi, and one Angelman cases (Fig. 3a,e,i, respectively) were confirmed. The MS-HRM analysis performed using the Qiagen-DBS extraction method detected 102 healthy individuals (Fig. 3b). Altogether, the MS-HRM analysis on Mem-DBS and Chellex-DBS samples detected, respectively, 82 and 94 healthy patients (Fig. 3c,d). In addition, the dissociation curve analysis of the DNA obtained from DBS by the three methodologies confirmed all 20 individuals with PWS by the absence of the paternal peak (Fig. 3f–h), and one individual with AS without maternal methylated allele peak (Fig. 3j–l).

## Discussion

The clinical diagnosis of PWS and AS in newborns is challenging since the distinctive phenotypic characteristics of the diseases are not evident during this phase. Reliable and low-cost molecular analysis techniques are imperative for accurate and early diagnosis to start precise treatment. Our group described an MS-HRM methodology using a unique pair of primers to evaluate the DNA methylation pattern of the exon 1-promoter region of the *SNURF-SNRPN* gene^16^. This approach makes it possible to distinguish paternal and maternal alleles according to a clear difference in melting temperature. However, other DNA methylation-sensitive techniques (such as MS-PCR and MS-MLPA) also use DNA extracted from the WB sample and require minimum logistics for collecting, preserving, and transporting blood samples within a time frame to preserve its integrity until delivery to a specialized diagnostic center^26^.

**Figure 4:**
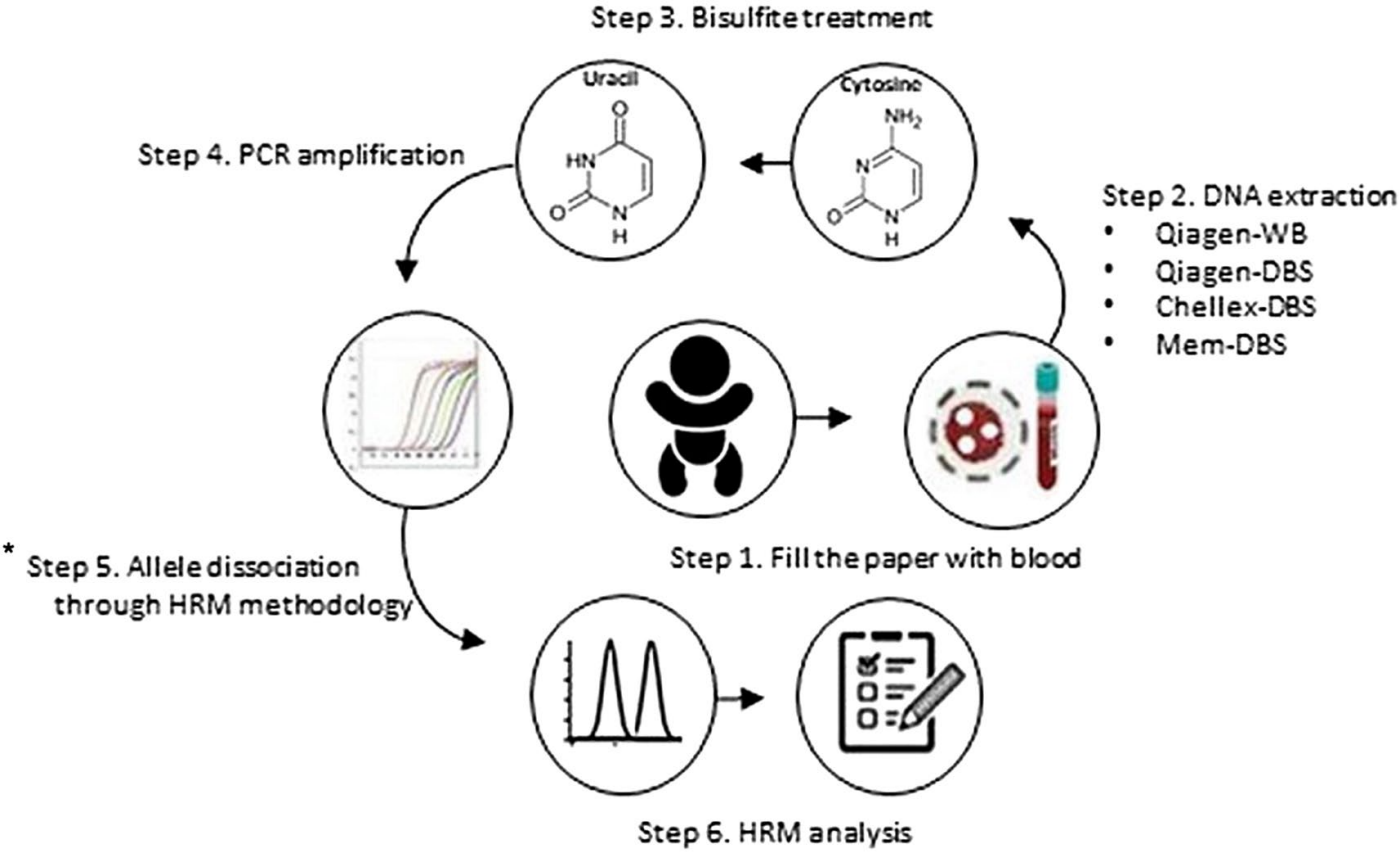
Methodology of DNA methylation analysis for DBS. Step 1: Collect blood and fill the spots on filter papers; Step 2: Extract the DNA from each Guthrie cards and from Whole Blood; Step 3: Bisulfite treatment, converting unmethylated cytosine to uracil; Step 4: PCR amplification; Step 5: Allele discrimination through HRM methodology: Step 6: Results analysis and diagnostic.*It does not distinguish between the different molecular causes related to PWS and AS.

This study evaluated different methodologies for DNA extraction from DBS to screen PWS/AS using the MS-HRM method (Fig. 4). Guthrie cards are easy to store and ship from isolated areas to diagnostic centers, and they represent a reliable platform for the accurate and fast diagnosis with a small amount of blood^21^. Our study tested three different DNA extraction methods: Qiagen-DBS, Mem-DBS, and Chellex-DBS in comparison with DNA extracted from WB (Qiagen-WB). Initially, we observed a significantly higher DNA concentration with Mem-DBS and Chellex-DBS methodologies compared to Qiagen-DBS. However, the Mem-DBS and Chellex-DBS parameters for 260/280 and 260/230 indicated lower DNA purity, suggesting the presence of contaminants such as phenol and proteins. The Qiagen-DBS method used to extract DNA from DBS showed a significantly higher DNA quality and purity compared with Mem-DBS and Chellex-DBS. As previously reported, better quality DNAs are more appropriate for molecular biology activities^27^.

In order to detect PCR inhibitors for each DBS-DNA extraction method, the reference control gene *RPP38* was used as an amplification internal control. Even though *RPP38* was amplified by all DNA extraction methods, Mem-DBS and Chellex-DBS displayed higher Ct values when compared to Qiagen-DBS. The lower Cts values observed after Qiagen-DBS DNA extraction likely relate to better DNA integrity and purity, as previously reported^28^.

The MS-HRM results from 123 samples by Qiagen-DBS, 103 by Mem-DBS, and 115 by Chellex-DBS DNA isolation methods indicate an amplification efficiency of 98,4%; 82,4%; 92%, respectively. These results from DBS samples correlate with DNA purity. In addition, it has been shown that bisulfite treatment reduces considerably the DNA quality, contributing to reduced qPCR efficiency in all DNA extraction methods^29–31^.

The MS-HRM analysis from genomic DNA extracted from DBS displayed no significant difference from WB samples in terms of dissociation temperatures, suggesting that *SNURF*-*SNRPN* CpG methylation sites are preserved on DBS samples. This data was compatible with all DNA extraction methods that detected correctly all 20 PWS and 1 AS evaluated here. However, the HRM peaks related to the temperature of allelic dissociations were better represented with Qiagen-DBS methodology, suggesting that amplification quality is directly associated with better allelic dissociation peaks.

Despite the concordance in detecting all PWS and AS individuals, the efficiency to detect healthy individuals varied. The DNA extraction from DBS by Qiagen-DBS detected 102 Healthy individuals. On the other hand, the genomic extraction from DBS by Mem-DBS and Chellex-DBS detected 82 and 94 Healthy individuals, respectively. Our results indicate that once amplification of the bisulfite modified DNA occurs there is a clear dissociation curve that prevents misinterpretation. Our study also demonstrated that preferentially no amplification was seen on healthy individuals samples that represent only 1.6% of all samples tested. The amplification rate of the DNA obtained by Qiagen-DBS (98.4%) is compatible with other qPCR screening methods studies, where the efficiency varied from 48 to 100%^32–38^. The rate of amplification failure observed in Qiagen-DBS can decrease according to a new DNA extraction or new sample request. Despite this comparative analysis between the results obtained in this study and the results observed in the literature, it is difficult to compare the DNA from DBS amplification efficiency across different studies due to many reasons: different protocols of genomic extraction from DBS, diversity of commercial Guthrie Cards, storage conditions, and year-durations.

An earlier and accurate diagnostic provides not just the anticipation of drug administration but also other benefits, significantly reducing hospitalization and comorbidities. This is clear for children displaying the worst speech and language problems, commonly seen in patients with prolonged tube feeding^39^. The availability of an accurate and reliable technique to diagnose PWS and AS, especially for hypotonic neonates potentially could help to identify and treat those patients^40^. The MS-HRM analysis associated with DBS samples provides a platform for neonatal screening using molecular techniques, even in remote areas.

The possibility of massive and accurate screening diagnostic methodology of newborns for genetic diseases also affects the routine application of public health services. Several syndromes require periodic clinical surveillance, and the distinction between severe and milder syndromes reduces costs with genetic disorders misdiagnosed. Novel diagnostic tools to improve neonatal diagnostics will direct newborns to disease-specific government programs with specialized multidisciplinary teams that ultimately leads to better prognosis and quality of life^41^.

Recently, some molecular studies using DBS as a DNA source have been performed^37,38^. DBS provides only a small amount of DNA^42^, however, it was demonstrated to be adequate for MLPA analysis and diagnosis of 22q11 deletion syndrome (22q11 DS) according to Copy Number Variations (CNV)^43^. The gold standard method for the diagnosis of 22q11 DS is the FISH technique using whole blood. In the same way, FISH is also used for PWS testing and can detect 15q11-q13 deletions. The possibility to use DNA extracted from DBS in different diagnostic methodologies could facilitate the detection of each genetic mechanism related to PWS. DBS is commonly used as a screening method for disorders in newborns, such as Gaucher, Pompe, Fabry, and Mucopolysaccharidosis-I^44–46^. The use of DBS as a source of DNA enables the massive screening of severe diseases in newborns, where early diagnosis allows effective treatments. For instance, the Severe Combined Immunodeficiency (SCID) is a heterogeneous group of genetic diseases characterized by severe T cell lymphopenia with often lethal outcomes due to late diagnosis^47^. T Cell Receptor Excision Circle (TREC) quantitative analysis from DBS by qPCR has shown to be a powerful and economical methodology for detection of SCID in newborns, providing an early and life-saving treatment^48^.

MS-HRM is a robust methodology for laboratory diagnostic and research use. Charoenkwan et al.^49^, established a pattern of High-Resolution Melting curve to detect genetic variations related to beta‑thalassemia disease with no need for traditional DNA sequencing. Due to the high sensitivity of the MS-HRM technique, single nucleotide polymorphism (SNP) can be identified in a DNA fragment^49^. The gold standard method to analyze mutations in DNA is Sanger sequencing, which is time-consuming with several steps and laborious^50^. The use of HRM methods to detect sequence variations from DBS opens the possibility to develop specific assays to newborns screening for disease-related mutations impacting neonatal development.

## Conclusion

The MS-HRM analysis to screen PWS and AS associated with DNA extraction from DBS achieved 100% of concordance compared to MS-HRM performed with traditional whole blood methodology. The use of the DBS sample as the main source of DNA provides several advantages against the use of WB; demanding only a small amount of blood, less invasive procedure with a considerable reduction of the risk of contamination, ease of storage, and transportation. Furthermore, central laboratories can analyze DBS from remote areas, avoiding geographic barriers, and allowing long term storage. This method showed accuracy and no misinterpretation was observed in our experience. We recommend the MS-HRM molecular screening tests preferentially for hypotonic neonates in order to anticipate diagnosis and improve prognosis. Given the widespread use of DBS as a neonatal screening method, the MS-HRM analysis from this sample does not require new facilities or guidelines. Our study demonstrates the potential of DBS as a DNA source for MS-HRM studies and its accuracy on abnormal DNA methylation detection of imprinting related disorders.

## Methods

### Sample collection

The study was approved by the Fernandes Figueira Institute IRB (CAAE: 45767015.0.0000.5269). Guthrie cards were obtained from babies born at Fernandes Figueira Institute from mothers enrolled randomly during our regular prenatal follow up. No selection criteria were used to our sample should reflect the general population. Our study period comprised 1 year and was able to access 125 stored Guthrie cards filled with a drop of peripheral blood from neonates. Of these 125 stored DBS cards, we had 45 additional whole blood fresh samples which were also used as positive controls (20 PWS, 1 AS, and 24 healthy control patients), and further used for comparative purposes analysis between DBS extraction methods.

### DNA extraction protocols

Genomic DNA isolation from DBS cards was performed using single-hole paper punches each 3.2 mm in diameter using three different protocols:

1. Qiagen-DBS: DNA isolation was performed with the QIAamp DNA Mini kit (QIAGEN, Germantown, MD, USA) following the manufacturer’s protocol. Briefly, the DNeasy column-based isolation method started with three paper punches being incubated with proteinase K for 3 h with shaking at 56 °C. Then, two elutions were performed, and each time 20 μL of LoTE buffer (low tris-ethylene diamine tetraacetic acid) was used (Qiagen-DBS). The same DNA extraction method was performed with the peripheral whole blood from Prader-Willi, Angelman, and healthy control patients (hereby called Qiagen-WB).
2. Mem-DBS: The Mem heat extraction protocol was performed as described by Barbi et al. (1996) using three paper punches from the Guthrie cards (Mem-DBS)^24^.
3. Chellex-DBS: For our third DNA extraction, three paper punches from DBS were washed with 1X PBS/0.1% Tween-20 for 10 min and transferred to a new 1.5-mL microcentrifuge tube, containing 60 μL of nuclease-free water. After that, 10 μL of Chelex-lysis solution were added, following incubation for 30 min at 60 °C and another for 30 min at 95 °C. The Chelex was pelleted at 20,000 g for 1 min, the supernatant was discarded and the microcentrifuge tube was storage at − 20°C^25^.

### DNA quantification

DNA concentration and purity (260/280 and 260/230 ratios) were assessed by NanoDrop 2000 Spectrophotometer (Thermo Scientific, Waltham, MA, USA) from DBS or peripheral whole blood samples processed by each of the three extraction protocols.

### Ribonuclease P (*RPP38*) amplification

To ensure DNA integrity and to exclude the possibility of false negatives due to the presence of eventual inhibitors, the TaqMan *RPP38* Control Reagents kit (Catalog number 4316844, Applied Biosystems, Foster City, CA, USA) was used as a reference amplification control following the manufacturer’s protocol. All reactions were performed in a MicroAmp Fast Optical 96-Well Reaction Plate using the 7,500 Fast Real-Time PCR System Mix (Applied Biosystems).

### Bisulfite treatment

A total volume of 20 μL [20 ng/μL] of DNA extracted from DBS and WB was treated with EZ-96 DNA Methylation-Gold Kit (Zymo Research, Irvine, CA, USA), following the manufacturer’s protocol. Bisulfite converted DNA was quantified by NanoDrop 2000 Spectrophotometer (Thermo Scientific).

### Methylation-sensitive high-resolution melting (MS-HRM)

The MS-HRM was performed in triplicates with the bisulfite-treated DNA isolated from DBS or WB from each individual. It was performed in a MicroAmp Fast Optical 96-Well Reaction Plate using the 7,500 Fast Real-Time PCR System Mix (Applied Biosystems) with the primers 5′‐GGATTTTTGTATTGCGGTAAATAAG‐3′ and 5′‐CAACTAACCTTACCCACTCCATC‐3′ (forward and reverse, respectively) as previously described^16^. The melting temperatures of 78 °C and 83 °C were chosen as a near-proportional amplification of unmethylated and methylated alleles, respectively. As described by Ferreira et al. (2019), the pair of primers used in this study act as a positive control for the bisulfite conversion, process due to the particularity of annealing in the treated DNA (Additional file 2: Figure S1).

### Statistical analysis

Each group analysis was done with the unpaired Student’s t-test to detect differences among them. A two-sided *P* value < 0.05 was considered statistically significant. Percentile, mean, median, and standard deviation values of *RPP38* amplifications were also calculated for comparative purposes.

### Ethics approval and consent to participate

The Fernandes Figueira Institute IRB approved the study (CAAE: 45767015.0.0000.5269). The written informed consent terms were obtained from all participants in this study and from the consent of the LAR or responsible for the minor involved. All experimental protocols in this manuscript were carried out in accordance with the ethical principles that govern research with human beings, in accordance with the guidelines of the Declaration of Helsinki.

## Supplementary information

Supplementary information 1.

Supplementary information 2.

## Data Availability

The datasets generated and/or analyzed during the current study are not publicly available due to the confidentiality and ethical aspects related to patient data but are available from the corresponding author on reasonable request.
