# Supplementary material for: A newborn screening pilot study using methylation-sensitive high resolution melting on dried blood spots to detect Prader-Willi and Angelman syndromes
Source: Sci Rep. 2020 Aug 3;10:13026. doi: 10.1038/s41598-020-69750-0 (PMC7400512; doi:10.1038/s41598-020-69750-0)
Supplement: Supplementary file 1 — Supplementary information 1. [file 41598_2020_69750_MOESM1_ESM.docx]

**Additional file 1**

**Title:** A newborn screening pilot study using Methylation-Sensitive High Resolution Melting on dried blood spots to detect Prader-Willi and Angelman syndromes

**Submission ID** 45cfe8d0-c1a1-4a8d-9ad1-0d95750071e6

**Authors:** Igor Ribeiro Ferreira, Régis Afonso Costa, Leonardo Henrique Ferreira Gomes, Wilton Darleans dos Santos Cunha, Latife Salomão Tyszler, Silvia Freitas, Juan Clinton Llerena Junior, Zilton Farias Meira de Vasconcelos, Robert D. Nicholls, Letícia da Cunha Guida

**Corresponding author**

Letícia da Cunha Guida, PhD

Instituto Nacional da Saúde da Mulher, da Criança e do Adolescente Fernandes Figueira, Fiocruz

Avenida Rui Barbosa 716, Flamengo, Rio de Janeiro/RJ, Brazil

ZIP Code: 22250-020

Telephone: +55 21 25541919

**Table S1 - Statistical information of *RPP38* amplification according to each DNA extraction method.**

|  | Qiagen-WB | Qiagen-DBS | Mem- DBS | Chellex-DBS |
| --- | --- | --- | --- | --- |
| Number of values | 45 | 80 | 80 | 80 |
|  |  |  |  |  |
| Minimum | 25 | 26 | 27 | 27 |
| 25% Percentile | 26 | 27 | 29 | 28 |
| Median | 27 | 28 | 29 | 29 |
| 75% Percentile | 27 | 29 | 30 | 30 |
| Maximum | 28 | 31 | 32 | 32 |
|  |  |  |  |  |
| Mean | 26,58 | 28,18 | 29,53 | 29,03 |
| Std. Deviation | 0,7121 | 1,184 | 1,141 | 1,359 |
| Std. Error of Mean | 0,1126 | 0,1184 | 0,1141 | 0,1359 |
|  |  |  |  |  |
| Lower 95% CI of mean | 26,35 | 27,95 | 29,3 | 28,76 |
| Upper 95% CI of mean | 26,8 | 28,41 | 29,76 | 29,3 |
