# Supplementary material for: A newborn screening pilot study using methylation-sensitive high resolution melting on dried blood spots to detect Prader-Willi and Angelman syndromes
Source: Sci Rep. 2020 Aug 3;10:13026. doi: 10.1038/s41598-020-69750-0 (PMC7400512; doi:10.1038/s41598-020-69750-0)
Supplement: Supplementary file 2 — Supplementary information 2. [file 41598_2020_69750_MOESM2_ESM.docx]

**Additional file 2**

**Title:** A newborn screening pilot study using Methylation-Sensitive High Resolution Melting on dried blood spots to detect Prader-Willi and Angelman syndromes

**Submission ID** 45cfe8d0-c1a1-4a8d-9ad1-0d95750071e6

**Authors:** Igor Ribeiro Ferreira, Régis Afonso Costa, Leonardo Henrique Ferreira Gomes, Wilton Darleans dos Santos Cunha, Latife Salomão Tyszler, Silvia Freitas, Juan Clinton Llerena Junior, Zilton Farias Meira de Vasconcelos, Robert D. Nicholls, Letícia da Cunha Guida

**Corresponding author**

Letícia da Cunha Guida, PhD

Instituto Nacional da Saúde da Mulher, da Criança e do Adolescente Fernandes Figueira, Fiocruz

Avenida Rui Barbosa 716, Flamengo, Rio de Janeiro/RJ, Brazil

ZIP Code: 22250-020

Telephone: +55 21 25541919


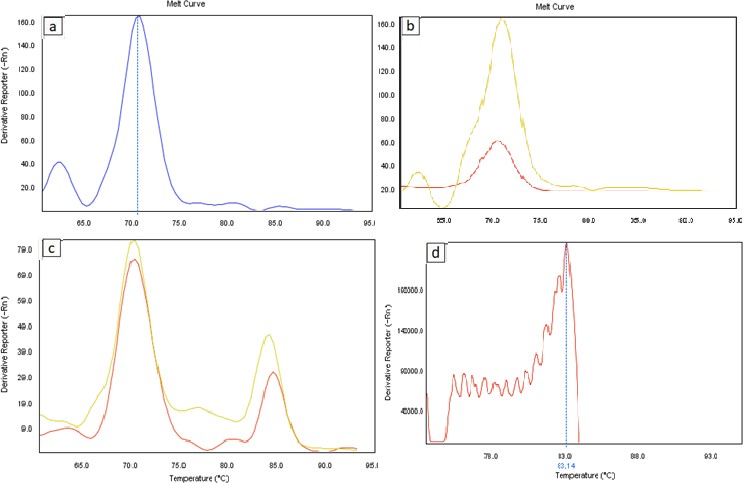


**Figure S1: Analysis of the HRM curve of the DNA of a patient with converted and unconverted SPW with bisulfite treatment.** In (a) Negative control (without DNA), (b) Non-converted DNA with treatment, (c) Mixing 50/50% of converted and non-converted DNA, and (d) converted DNA. When comparing with the control result (a), we observed that the peak that appears in (b) is the lowest temperature peak in (c) corresponds to the primary dimers. In (d), we have the fusion pattern compatible with that of a person with Prader-Willi syndrome (SPW). The different colors correspond to the same samples in experimental replicates
